# Analysis of Consumer Behaviour in the Context of the Place of Purchasing Food Products with Particular Emphasis on Local Products

**DOI:** 10.3390/ijerph20032413

**Published:** 2023-01-29

**Authors:** Agnieszka Dudziak, Monika Stoma, Emilia Osmólska

**Affiliations:** Department of Power Engineering and Transportation, Faculty of Production Engineering, University of Life Sciences in Lublin, 20-612 Lublin, Poland

**Keywords:** consumers behaviours, food products, place of purchasing, local food, ethnocentrism, sustainable consumption

## Abstract

Background: Researchers and marketing specialists study consumer behaviour in the market because it is an important part of economics. There is a growing trend among consumers to buy local products. Consumers know that buying local products is seen to support local producers and entrepreneurs and protect the domestic economy. Methods: A diagnostic survey analysis was conducted among 404 respondents from Eastern Poland in 2022 to achieve the research objectives. The aim was to present the specifics of the phenomenon of consumer behaviour in relation to the place of purchase of food products, with particular emphasis on local products, according to the qualitative approach adopted. An attempt was made to identify and evaluate the attitudes and declarations of individual consumers in this area. A questionnaire was used as the research tool, and its distribution was carried out entirely electronically via the Internet. Results: The study showed that shopping habits vary by location and age, with hypermarkets and local stores popular among both men and women, while online shopping and wholesale options were also common. Conclusions: Understanding how consumers make decisions is a complex task, as it involves various factors such as thoughts, emotions and actions that lead to product selection and purchase. These factors can vary based on demographic characteristics, such as gender, age, and location. Studies have shown that Polish consumers are more likely to buy local products from large chain stores than from smaller manufacturer-owned stores, and prefer domestically produced goods, which is reflected in an ethnocentric attitude toward the local market.

## 1. Introduction

Consumer behaviour in the market is an important element of economics, so it is studied by researchers and marketing specialists. There are many different theories and models describing consumer behaviour, but generally, people make decisions to purchase products or services based on many different factors, such as needs, preferences, budget constraints, and the influence of advertising and promotions [1,2]. In addition, consumers may also be influenced by factors such as loyalty to certain brands, recommendations from friends and family, and social influence and peer pressure. Some of these factors may be more or less important depending on individual consumers and the situation [3,4].

Therefore, the concept of 4P is an important element to consider. The 4P concept, also known as the “marketing mix,” is one of the basic tools of marketing management, which consists of four elements: product, price, distribution (also known as “place”), and promotion. These are factors that influence how a product is perceived by consumers and how well it is sold in the market [5,6,7]. 

Place of purchase, as an element of the marketing mix, refers to where a product is available to consumers [8]. This can include places such as different types of stores, as well as ways in which consumers can order it, such as through websites or via mobile apps [9]. The decision of where to buy the product is extremely important for consumers as it has a significant impact on their satisfaction with their purchase. It is also important to ensure that there are sufficient points of purchase for the product so that consumers can easily find and acquire it [10].

It should be added that contemporary consumers increasingly often decide to buy not only typical food products, but also local products. Local products are those that are produced or come from a specific geographic area, such as a city, region, or country [11,12]. They are often promoted as an alternative to imported or mass-produced products, and their purchase may be seen to support local producers and entrepreneurs and protect the local economy. Local products can include a wide range of products, such as food, drinks, handmade items, and many others [13,14,15]. They can be found in stores specializing in local products or at markets, as well as in grocery stores and other retail stores [16,17,18]. Many consumers have reoriented themselves towards local food, i.e., food that has travelled only short distances or towards food that is marketed directly by the producer [11].

Because the place of retail sale is particularly relevant for consumers, who make their final purchasing decisions and buy specific products there, the aim of this article is to identify and analyse the attitudes and preferences of consumers from Eastern Poland, and especially from the Lubelskie Voivodeship, regarding the places where they buy food products, with a particular emphasis on local products. To achieve this aim, intermediate aims were also set, namely the analysis of consumers’ statements about the perceived availability of local products. The research was based on three grouping variables: gender, age, and place of residence.

However, it seems that despite the local nature of the area and research sample, as well as its preliminary nature, the contribution of the research is significant. First, there is very little research in this region of Europe in the context of places of purchase for food products, considering at the same time the gender of consumers, in relation to their age and place of residence. In addition, the obtained results can be an important starting point for further in-depth considerations, especially in terms of respondents’ attitudes and statements about the purchase of local products, and consequently, the phenomenon of ethnocentrism towards the regional market. This may be crucial for the development of appropriate marketing strategies by various companies offering wholesale and retail sales of food products.

The article has been divided into the following sections: Section 2—literature review, Section 2.1—consumer behaviour in the context of the place of purchasing food products, Section 2.2—local food products, Section 2.3—consumer ethnocentrism, Section 3 —materials and methods, Section 3.1—study design—the choice of the research method and study area, Section 3.2—research problems, Section 3.3—survey questionnaire as a research tool, Section 3.4—research sample, Section 3.5—data analysis, Section 4—results and Section 5—discussion. Finally, Section 6 presents the conclusions of the analyses and deliberations carried out.

## 2. Literature Review

### 2.1. Consumer Behaviour in the Context of the Place of Purchasing Food Products

The implementation of research related to the lifestyle and character traits of consumers made it possible to see that decisions regarding the place of purchase and consumption are rarely made by consumers in a straightforward manner, as reported in the literature on consumer behaviour [11]. Consumer behaviour as a whole, including that related to the place of purchase, is affected by a number of factors, the sources of which can be found in the customer’s external environment, mainly in the retail environment [19].

In many situations, it is recognized that the most important determinant in terms of the success of commercial and service activities is location. When other elements (availability of goods and their price) are constant, consumers decide to shop in the store that is closest and offers the necessary goods. In addition, when possible, the consumer usually chooses the largest store that is located in the area [20]. 

Maciejewski [19] conducted an analysis of consumer preferences in relation to making purchases. They revealed that the decision to choose where and when to shop is mainly influenced by the type of product and how important it is in the process of meeting needs. According to the survey, food products are most often bought by consumers in discount stores. As many as 63% of respondents buy groceries in shops such as Lidl, Biedronka or Netto. Hyper- and supermarkets came second, but this choice was made by only 16.5% of consumers. 

Buying groceries at discount stores used to be a sign of low social status. However, it is now becoming fashionable. On the other hand, more than 30% of respondents decide to buy cleaning products at discount stores. Though, this is not a place where respondents prefer to buy such products. Most people decide to buy cleaning products in hypermarkets—about 40%. Discounters and hypermarkets are also not placing where consumers would be happy to shop for clothing, footwear, or household items of their choice. To buy these things, most of the respondents choose a specialist store. In addition, online stores are a popular point of purchase of these goods. Approximately 20% of respondents indicated that they shop in this way [19].

Nearly 90% of the surveyed consumers indicated that they visit shopping malls. They go there mainly for clothing and footwear (approx. 74%), food (61%), and cosmetics (60%). In addition, shopping malls offer a number of services that attract customers’ attention—these include: food and beverage (approx. 68%), culture (34%) and banking (27%) [19].

Referring to the purchases made in supermarkets, research conducted in the USA and Germany can be mentioned [21]. They showed that differentiation of the price and quality of food products has a significant impact on consumer purchasing preferences. Nevertheless, more research needs to be done to determine the impact of food prices on food purchases. Especially, as there is a trend that the lower the price and the greater the variety, the higher the sales of such products. 

**Figure 1:**
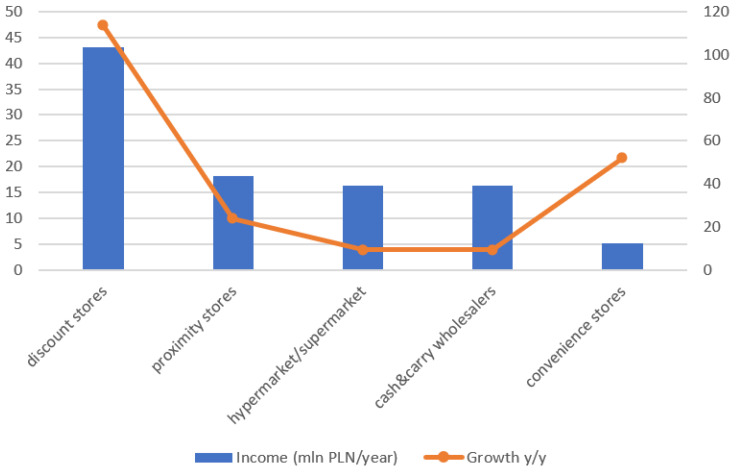
Revenue of various retail locations for 2020 [22].

It might seem that the pandemic crisis has a very negative impact on the revenues of various retail chains. The seasonal disruption was noticeable and looked significant, but analysing the data in the longer term, the unstable situation has evened out. The largest retail chains operating as discount stores had the highest revenue, reaching even 47.33 million PLN/year. Detailed data is presented in Figure 1.

In addition, the ongoing pandemic crisis from 2019, indicates radical changes in consumer behaviour. Survival psychology researchers have found that events such as natural disasters, healthcare crises and terrorist threats change consumer behaviour [23]. Furthermore, changes in the purchasing process during the pandemic include the emergence of a tendency to impulsively panic buy various products, sometimes unnecessary. All this has had a huge impact on shopping habits and purchasing decisions [24]. However, the location has a positive and significant impact on customer satisfaction and product loyalty [1,3,4].

In Poland, food products are sold primarily through traditional distribution channels. Modern channels, such as online food sales, are not very popular yet—they account for about 1% of the total. However, it should be added, that it is one of the fastest and most dynamically developing categories of online shopping [25].

With the development of the Internet and e-commerce, customers’ choice of where to shop may become a process consisting of not two, but three phases. In the first, they decide whether to leave home at all, or whether it is better to opt for a virtual outlet [25]. In the second stage, they decide on the shopping area (i.e., the location—outskirts of the city, city centre), and finally they decide on a specific shopping facility in the area [26]. 

It also happens that consumers moving around in space (on their way from work to home) notice stores and the goods displayed in them, which makes them want to go inside and make purchases. In this case, it can be said that there is a kind of “entry reflex” without a predetermined intention to buy anything. This is because such behaviour (entering the store, looking at the goods and buying) makes it possible to satisfy needs beyond ordinary rational and economic human needs. The consumer’s motivation also includes the search for entertainment and the desire to relax. These elements significantly hinder the purchasing model.

Research conducted in Poland and worldwide shows that in the case of virtual space, online stores are the most popular, with people participating in the survey usually choosing to buy books and multimedia (almost 46%) and clothes and shoes (about 41%). The second place in the virtual space in terms of purchasing the above-mentioned products was taken by online auctions. About 37% choose to buy clothes and shoes here, and 25% buy books and multimedia. Household goods, cleaning products, as well as software and computer hardware (apart from online stores) are purchased by respondents via price comparison websites, e.g., Skąpiec, Ceneo, and Okazje. When it comes to sports and leisure products, as well as beauty and healthcare, apart from online stores, group shopping websites, such as Groupon, are also popular [19].

It should be added that by deciding on a specific place to buy, the consumer purchases the whole product, which consists not only of the specific item in which he or she is interested, but also of the packaging, service, advertising, product images and the atmosphere of the place where the transaction takes place. The atmosphere is treated as one of the most important determinants of the purchasing process [27]. Atmosphere is not the objective physical and social elements that create the image of the store, but the subjective feelings aroused in consumers by these elements. Store owners shape the store atmosphere by paying attention to visual (lighting, colour, shape), olfactory (freshness, smell), auditory (tone colour, loudness) and tactile (temperature, softness) aspects. These factors influence consumers’ decisions regarding the choice of where to buy services and goods [2]. 

In summary, it should be stated that the primary task of retail trade is to create appropriate conditions for the purchase of products by consumers. Purchases should be made at a place and time suitable for the consumer, in accordance with the consumer’s desires and in circumstances that the consumer considers favourable [28]. 

### 2.2. Local Food Products

In recent years, it has been noticed that the topic of a healthy lifestyle and comprehensive and holistic healthcare is increasingly being addressed as a new consumer trend [29]. The consumption of regional or local products fits perfectly into this trend. Currently, the market of regional and traditional products is distinguished by its diversity. Therefore, traditional or local products should contain all the necessary information about the origin, quality and price of a given product [30]. 

The fashion for local products has developed along with the development of global brands. It should be added that the developed trends of global brands do not exclude tradition but complement modern consumption. Consumers are often looking for forgotten products, which in previous years enjoyed great recognition and a good reputation, created on the basis of old recipes [31], local or regional products.

Nowadays, the development of the production of local goods is extremely important because products of this type encourage consumers to promote their regions, and thus support the development of tourism and recreation in a given area, which in turn also activates the broadly understood service market. The expansion of the production of local products also leads to the development of the region, integrates society, protects tradition and provides the market with good quality products [12]. 

Furthermore, research conducted by Bryla [24] on the importance of country-of-origin information on food product packaging showed that 18.8% of respondents indicated the country of origin as a very important type on food packaging. Then 32.2% of respondents considered that the country of origin on the packaging was rather important, while as many as 35.4% considered it to be of medium importance, 10.5% considered it rather unimportant, and 3.1% considered it to be of medium importance, and 3.1% unimportant. Furthermore, as the author pointed out, even more research on this topic should be conducted due to the limitations that emerged during the analysis, i.e., cultural, political, and socio-economic differences.

The topic of local products is very important due to the changing consumer preferences. As mentioned above, consumers are increasingly looking for products that come from their country or region. However, it should be added, that consumer research conducted at the turn of recent years shows two directions of perceiving local products. One is based on processes that support [15] and protect the country’s economy [16]. It is also a practical way to protect the ecosystem. The second direction indicates that products from the region are natural, fresh, and safer than products from the global market [17].

The key issue is also what the concept of locality means. According to the researchers, it is important to define what the footnote “produced locally” means to consumers. Defining this concept will enable farmers to implement diversification of the products offered, which is aimed at increasing profit [18]. For example, in 2015, US farms generated a profit of USD 8.7 billion. The profit came from the sale of regional products [21]. What is very important, consumers choose such products because of safety, higher quality, lower consumption of fossil fuels and support for the country’s economy [32].

Hu et al. in their research [33] noticed that local products were more attractive to consumers, therefore there is a strong motivation to buy such food. Crucially, consumers perceive the impact of purchasing local products, which translates to suppliers, retailers, and the much wider community. Such behaviour is closely related to consumer ethnocentrism (Section 2.3. describes this phenomenon in more detail). Furthermore, some studies have shown that the main determinant of buying local food is this phenomenon. Consumers believe that if they do not buy domestic food, they will deprive people of jobs, resulting in losses in the economy [13,16].

### 2.3. Consumer Ethnocentrism

Ethnocentrism is a belief in the superiority of one’s own culture or ethnic group over all others. It is the tendency to judge other cultures based on the standards and values of one’s own culture. Ethnocentrism can lead to a lack of cross-cultural understanding, prejudice, racism and discrimination [34,35]. 

When it comes to consumer behaviour, ethnocentrism can affect in many ways. Consumers may be reluctant to buy products made in other countries or may be more inclined to buy products from their own culture. Consumers may also be more likely to patronize stores or services that are tailored to their own cultural group. Additionally, ethnocentrism can lead to a lack of understanding of different cultures, which can lead to different stereotypes and prejudices [31,36].

Moreover, ethnocentrism can also lead to a lack of diversity in the market. Consumers may be less likely to buy products from a company that does not reflect the values of their own culture. This can lead to a lack of competition and choice in the market. In addition, as already mentioned, ethnocentrism can lead to a lack of understanding between cultures, which may consequently initiate various kinds of misunderstandings and conflicts. Therefore, it is important for companies to be aware of the potential impact of ethnocentrism on consumer behaviour and strive to create a diverse and inclusive market [37,38].

For example, Hanus [39] studied the phenomenon of ethnocentrism among Poles in terms of eating habits as a possible determinant of the development of short supply chain. A total of 660 people took part in the study. It turned out that ethnocentric attitudes, when it comes to food products, are more frequently observed due to the belief in the higher quality and safety of regional products. Such a belief is expressed by as many as 77% of the respondents. Moreover, as research shows, ethnocentrism manifests itself much more often among older consumers. This is probably due to their patriotic attitudes and more conservative beliefs. Therefore, in order to cope with the changes in consumer attitudes (fresh, local food, safety, no added preservatives), farmers and producers need to work closely together, creating short supply chains, i.e., from farmer to table [40].

In addition, research conducted by Szabuniewicz and Majkut [41] on consumer ethnocentrism among Polish consumers in the era of the coronavirus (COVID-19) pandemic has shown that Poles have very little knowledge of whether a given brand of products belongs to a Polish owner. Respondents indicated brands that had been sold to foreign entities for a long time, e.g., Wedel, Soplica, Winiary, Pudliszki, and Tyskie, as belonging to Polish entrepreneurs, while brands such as Gino Rossi, Wittchen, and Reserved were not indicated as Polish. Only educated women presented greater knowledge on this subject, which proves that it is still women who supply households and seek information about the best products, in terms of price, quality, and health, as well as about the place of origin of the goods. This indicates the need to include the education of Polish consumers in strategic plans and the use of national symbols and packaging of preferred Polish products, as well as Polish-sounding brands, chosen especially by people over 50.

It is worth mentioning that ethnocentrism can lead to a lack of innovation in product development. Consumers may be less likely to purchase products that are unfamiliar to them or that do not reflect their own cultural values. Companies may be less willing to invest in developing products that could benefit from different cultural perspectives. Therefore, it is important for companies to be aware of the potential impact of ethnocentrism on innovation and to embrace cultural diversity when developing new products and services. Furthermore, companies should strive to create a market where consumers can feel comfortable buying goods and services from different countries [41].

## 3. Materials and Methods

### 3.1. Study Design—The Choice of the Research Method and Study Area

To achieve the assumed research objectives in 2022, the analysis was conducted using the method of diagnostic survey on a group of 404 respondents from Eastern Poland. The aim was to illustrate the specificity of the phenomenon of consumer behaviour in the context of the place of purchase of food products, with particular emphasis on local products, according to the adopted qualitative approach. Therefore, an attempt was made to identify and evaluate the attitudes and declarations of individual consumers in this area. 

The main justification for choosing this form of analysis was the possibility of obtaining information about the subjective and objective behaviour of respondents by collecting a large amount of various data relating to the topics covered by the study.

Research on declared places of food purchases, as well as on the perceived availability of local products, varies from country to country, but also in different regions of a country. These differences may result from different definitions and methodologies used by researchers, but also from different traditions, habits, and behavioural or cultural norms observed or in force in a given area. Therefore, it seems, that information on the behaviour declared by consumers in the context of places of purchasing food products, including local products, is usually specific to a given area [18,42,43,44]. Hence, in order to obtain the most reliable results, research should be conducted on a micro scale. The choice of this type of research may also be supported by the fact that large scale national studies may be costly or may overlook differences in consumer behaviour due to regional differences and consequent consumption choices [45,46].

Therefore, the region of Eastern Poland was selected for the study, with emphasis on the Lubelskie Voivodeship, as this region is one of the 20 poorest regions in the EU [46,47]. It seems that this may be important in the context of the choice of places to purchase food products, as the inhabitants of the Eastern Poland region have relatively lower incomes than inhabitants of central or western Poland, among others, which may result in a higher share of expenditure on food in relation to other types of products or services. Ultimately, this may result in greater consideration and more in-depth analysis when choosing a particular place to purchase food products.

It should be added that Eastern Poland is a region with less industry and urbanization intensity; while there is a particular emphasis on agriculture [48]. This may also affect the choices made by consumers in the context of specific places of purchasing food products, as well as lower orientation regarding the availability of local products in specific locations, as products defined as local products are often the basis of the diet of these residents, due to their cultivation in fields or even in home gardens.

### 3.2. Research Problems

Given the stated aim of the research, the following research problems were posed:

1. Do food shopping destinations differ according to consumers’ age and place of residence?
2. Is food shopping via online platforms and stores mainly the domain of young and middle-aged people, especially those living in large cities?
3. Does the gender of the respondent impact perceptions of the availability of local products?
4. Is the age of respondents a significant variable in the perception of availability of local products?
5. Does place of residence affect perceptions of availability of local products?

### 3.3. Survey Questionnaire as a Research Tool

A structured survey questionnaire was used as the research tool, which was distributed entirely electronically via the Internet. The survey was completely anonymous, and the respondents themselves were aware of this. They were presented with the assumptions and purpose of the study. Their decision to complete the questionnaire was voluntary. The use of this form of data acquisition made it possible to obtain answers on a selected topic in a relatively short period of time, depending on the age group, place of residence or place of shopping. It should be added that the use of the questionnaire as a research tool also allows for the appropriate processing of the data obtained [49,50].

The questionnaire used in the study consisted of two parts—metric and substantive. The metric part of the questionnaire made it possible to obtain the necessary data about the respondents in the context of further characteristics of their socio-demographic characteristics. The group of differentiating variables included descriptive parameters of the respondent, such as gender, age, and place of residence. The grouping variables aimed to differentiate the respondents in more detail and additionally and showed differences in consumer perceptions of the problem of purchasing food products, including local products. In turn, the second part of the substantive questionnaire allowed for the provision of information on the posed research problems. It contained closed-ended, single- or multiple-choice questions. In some questions, it was also possible to add your own answer if none of the proposed options fully reflected the attitudes, behaviour, or feelings of the respondents.

Respondents were asked where they mainly purchase food, and where they think local products are available.

### 3.4. Research Sample

It was assumed that the research sample would include at least 350 units—inhabitants of Eastern Poland, consumers of food products. One of the non-random methods of research sample selection was applied—purposeful sampling. The rationale for choosing this type of method was that the research participants met the criteria defining the categories of grouping variables contained in the metric part of the questionnaire used in the study—this means a deliberate choice of the participant due to his characteristics [51]. It should also be added, that purposeful selection of the research sample is useful especially when randomization is impossible, e.g., when the population is very large—so it is used in qualitative research to identify and select information-rich cases in order to make the most appropriate use of available resources [52]. It can also be used when the researcher has limited financial, human, and organizational resources. An additional justification for choosing this sampling method arises when the study does not aim to generate results that will be used to generalize to the whole population [53,54].

In the research sample, the distribution of respondents by gender was varied, as there were 284 women (70.3%) and 120 men (29.7%), which means that more than twice as many women took part in the survey. This may be because in Poland, grocery shopping is still done mainly by women. The largest group of respondents in terms of age was young people aged 19–25 years, with 300 people, accounting for 74.3% of all respondents; the other groups were respectively: 26–40 years old—66 people (16.3%), 41–65 years old—26 people (6.4%), over 60 years old—12 people (they represented 3% of the respondents).

As for the last metric variable, i.e., place of residence, the highest number of respondents were living in rural areas—178 respondents (44.1%), residents of cities with up to 100,000 inhabitants—52 people (12.9%), residents of cities with 100,000–300,000 inhabitants—60 people (14.8%), while residents of the largest cities—over 300,000 inhabitants—accounted for 28.2% of the total number of respondents (114 people).

As for the distribution of respondents, the obtained structure may result from the fact that the study was conducted via the Internet. It is well known that young people are the main users of the Internet, so it is not surprising that they were the most numerous. It is worth noting that the research will continue, and the sample will be balanced with other categories of respondents in the future to study more equal groups of survey participants.

### 3.5. Data Analysis

The software Statistica 13.3. and Excel 2007 was used to compile the results of the study. Graphical as well as descriptive methods were used to present the results obtained.

To separate groups of respondents who showed similar food shopping habits, in the first step of the adopted research procedure, the dependent variable analysis method was used and presented by means of categorized histograms. These related to the place of daily shopping according to the gender and age variable.

Then, due to the choice of a qualitative approach in this study, it was necessary to use appropriate methods so that the results of the qualitative analysis would allow—on the one hand, for an unambiguous and on the other hand—for a reliable interpretation of the phenomena and the attribution of meaning to the statements of the participants in the study. For this purpose, one of the methods of statistical data analysis was used—the classification tree method for female and male shopping rules.

The CART (Classification and Regression Trees) method was used to classify the dataset. This is a nonparametric discrimination method that is gaining increasing popularity in research where the growth in information and data volume has resulted in a sudden increase in the need to analyse them [55,56]. The purpose of this method is to group and divide objects based on distinguished characteristics. It enables the automatic search for patterns and relationships in large data sets, organizing them into concise models. As a result, data mining techniques have quickly found application in marketing data analysis, including in segmentation studies and market selectivity studies [56,57].

The CHAID algorithm is one of the oldest classification tree methods, proposed by Kass [58]; according to Ripley [59], the CHAID algorithm is a successor to the THAID algorithm developed by Morgan and Messenger [59]. The acronym CHAID stands for Chi-squared Automatic Interaction Detector. The CHAID algorithm builds non-binary trees (i.e., trees in which nodes can have more than two branches) using a relatively simple algorithm that is particularly suitable for analysing large data sets. It is named after the basic (non-binary) tree-building algorithm, which in classification problems (where the dependent variable is naturally qualitative) relies on the chi-square test as a criterion for determining the next best split at each step. In regression problems (continuous dependent variable), the programme calculates the value of the F-test. To begin with, qualitative predictors are created from quantitative ones by dividing the distribution into a certain number of categories so that each contains roughly the same number of observations. For qualitative predictors, the classes are already defined in a “natural” way [60,61].

The CHAID algorithm often produces effective multivariate tables (e.g., when the dependent variable with multiple classes is being classified and the independent variables are also qualitative with multiple classes) [59,62]. As a result, it is popular in marketing and market research in the context of market segmentation studies. Both the CHAID and the C&RT methods create trees in which each node contains a split condition, to optimize prediction (of a quantitative dependent variable) or classification (of a qualitative dependent variable). Both types of algorithms can be used for regression and classification problems [63]. 

In addition, box plots of the availability of local products among female and male responses by gender and place of residence were generated to highlight differences in shopping.

Table 1 presents basic information on research, methods, and tools as well as sample selection criteria, which were implemented for the purposes of this publication. 

## 4. Results 

To realize and verify the established research problems, the places where respondents make their daily food purchases were analysed in the following section. In order to deepen the analysis, various differentiating variables were applied to consumers, such as gender, place of residence and age, which were included in the metric part of the survey questionnaire.

**Figure 2:**
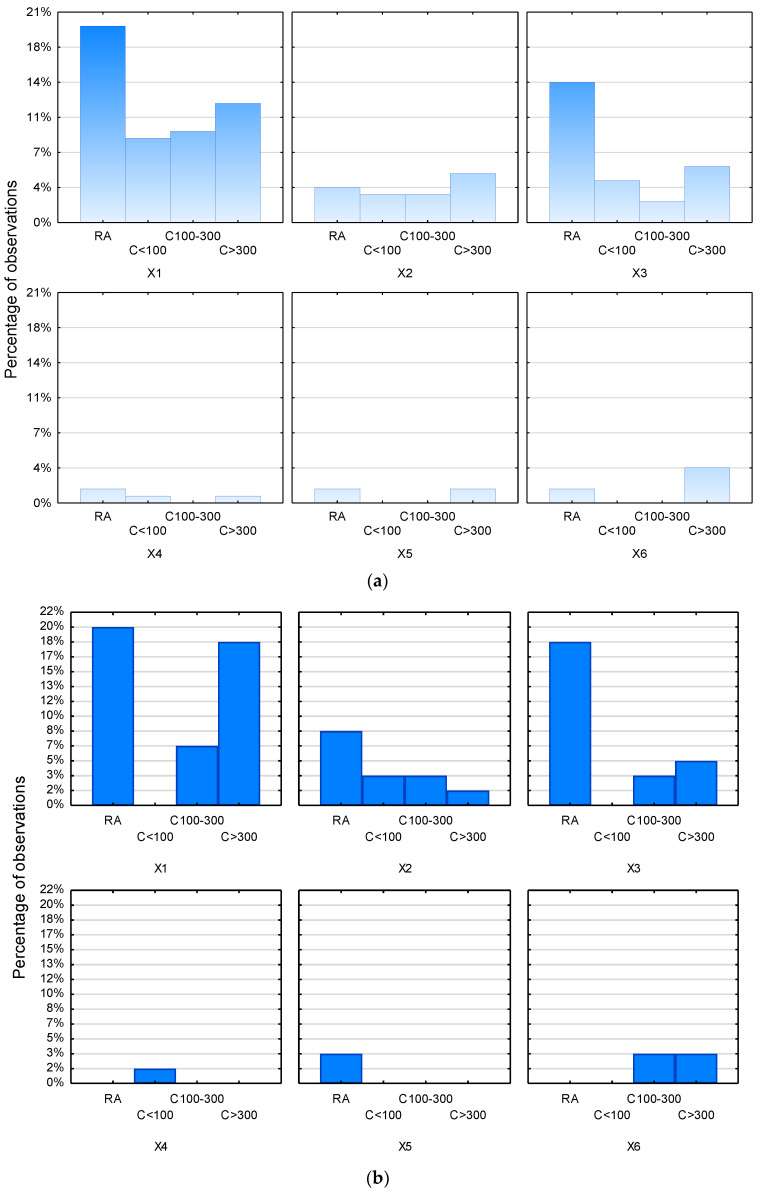
(**a**). Places of women’s daily food shopping. Histogram categorized by the variable place of residence. (**b**). Places of men’s daily food shopping. Histogram categorized by the variable place of residence. Abbreviations: X1-hypermarket, X2-via internet, X3-local store, X4-wholesale (cash and carry wholesalers), X5-armer, X6-marketplace, RA-rural, C100-city with up to 100,000 residents, C100–300-city with 100,000–300,000 residents, C > 300-city with more than 300,000 residents.

The data presented shows that both women and men declared and were most likely to choose hypermarket (X1), via internet (X2), local store (X3) as a place for daily shopping. The results are presented in a categorized histogram in Figure 2a (for women) and Figure 2b (for men).

Regarding the group of women (Figure 2a) considered by place of residence, it can be observed that women living in rural areas mainly purchased food products at hypermarkets and local stores. However, it should be added that sometimes they also chose other forms and places to purchase food products, such as online shops, local markets, cash and carry, or they buy these products directly from farmers.

Women living in small cities (up to 100,000 residents) also mainly chose hypermarkets and local store, but they also shopped online. For women living in medium-sized cities of 100–300,000 residents, answers such as hypermarket, online shopping and local store were indicated. It should be added that women living in small and medium-sized cities did not indicate local markets or farmers at all as places to purchase groceries. Furthermore, only women living in small towns declared that they also shopped at cash and carry, but not very often.

In contrast, women living in large cities (more than 300,000 residents) mainly shopped at hypermarkets, local stores, marketplaces, but also online.

However, in the context of the group of men group (Figure 2b) by place of residence, men living in rural areas purchased groceries mainly at hypermarkets and local stores.

Men living in small towns (up to 100,000 residents) mainly chose online shopping and wholesale (cash and carry wholesalers). Interestingly, they did not indicate supermarkets and local stores as places to purchase food products at all. The same applies to shopping at local markets or directly from farmers.

In the case of men living in medium-sized cities with a population of 100–300,000, responses such as hypermarket, via the Internet, local store but also marketplace were indicated. Men in this group did not at all declare buying food products at cash and carry shops or directly from farmers.

In contrast, men living in large cities (more than 300,000 residents) mainly shopped at hypermarkets, local stores, and marketplaces, although they also sometimes made purchases online.

**Figure 3:**
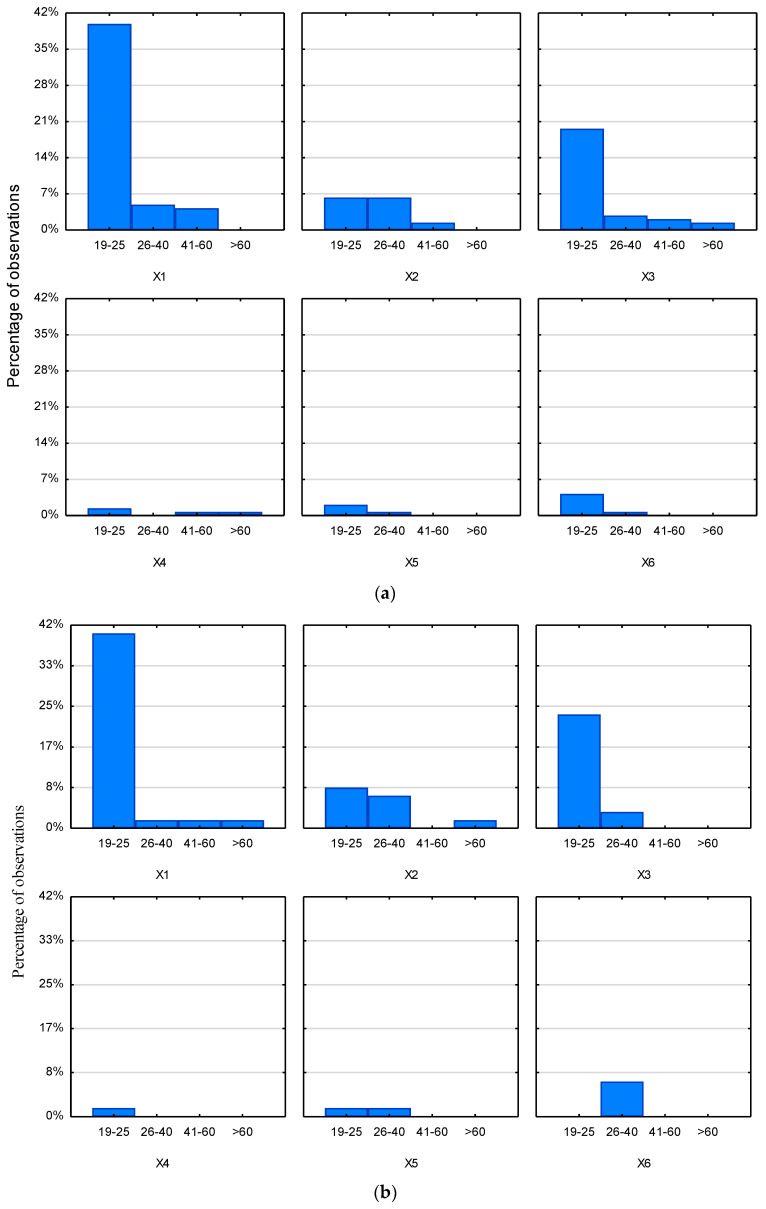
(**a**). Places of women’s daily food shopping. Histogram categorized by the variable age. (**b**). Places of men’s daily food shopping. Histogram categorized by the variable age. Abbreviations: X1, hypermarket; X2, via internet; X3, local store; X4, wholesale; X5, farmer; X6, marketplace; 19–25 years; 26–40, 26–40 years; 41–60, 41–60 years; >60, more than 60 years.

Regarding the women’s group (Figure 3a) by age, it can be observed that young women (19–25 years) mainly shopped for groceries in hypermarkets and local stores but also online and at marketplaces. Women aged 26–40 years mainly chose hypermarkets and online shopping, and to a lesser extent local store. Women in this age group did not indicate cash and carry shops at all as a place to do their food shopping.

For women aged 41–60 years, it was mainly hypermarkets, and to a much lesser extent, local stores, online shopping or cash and carry shops. Women in this age group did not report buying food products at local markets or directly from farmers.

In contrast, women over 60 years mainly shopped at local stores, but also wholesale stores (cash and carry wholesalers).

In the context of men’s daily food purchases, analysed regarding their age (Figure 3b), it should be noted that young men (19–25 years) bought groceries mainly at hypermarkets and local stores but also via the Internet. On the other hand, they shopped at cash and carries and directly from farmers with slightly less frequency. They did not declare making purchases at local markets.

Men aged 26–40 years mainly chose to shop at marketplace, and online, but also at local stores. In contrast, shopping in supermarkets was less popular among men in this age group.

In the case of men aged 41–60 years, these purchases were made mainly at the hypermarket. In contrast, men over 60 years made their food purchases at a hypermarket and online.

In the further part of the study, in the context of the purchases made by consumers, the demographic and social characteristics of the respondents were also verified in relation to the dependent variable (the dependent variable is the place where consumers make their purchases most often).

**Figure 4:**
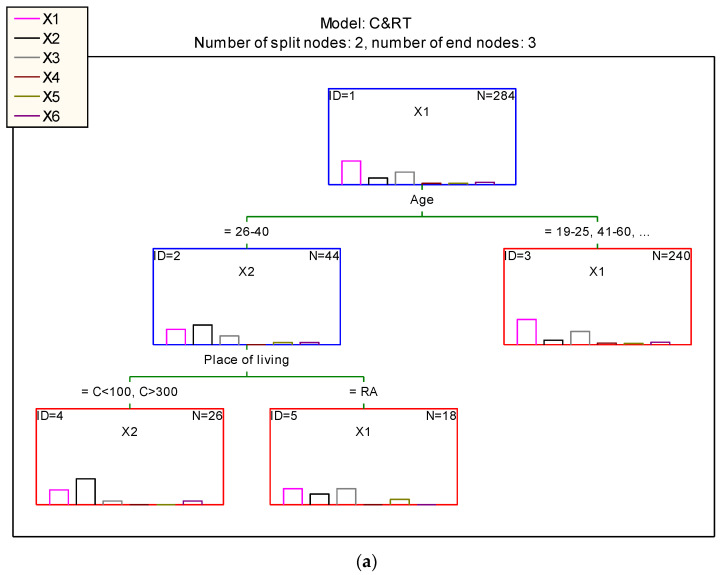

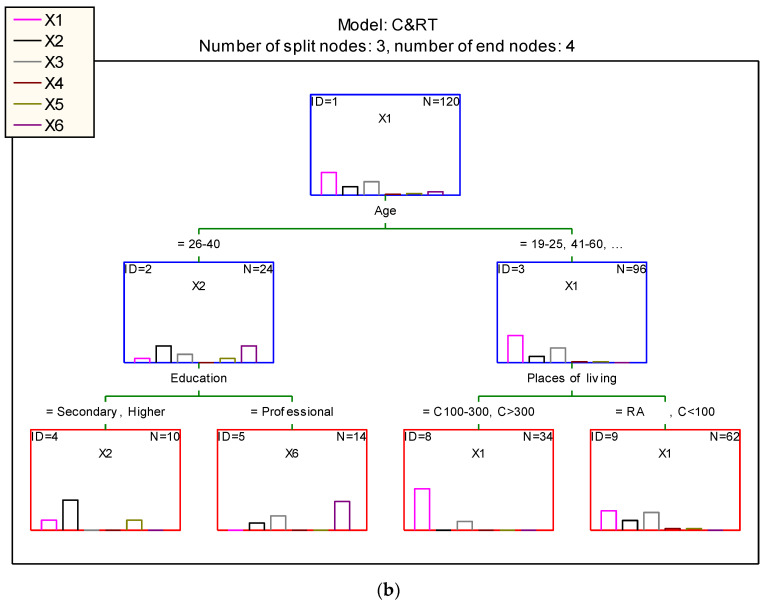
(**a**). Classification trees for rules regarding purchases by women. Abbreviations: X1-hypermarket, X2-via internet, X3-local store, X4-wholesale, X5-farmer, X6-marketplace, Age: 19–25 years, 26–40 years, 41–60 years, more than 60 years, place of living: RA-rural, C100-city with up to 100,000 residents, C100–300-city with 100,000–300,000 residents, C > 300-city with more than 300,000 residents. (**b**). Classification trees for rules regarding purchases by men. Abbreviations: X1-hypermarket, X2-via internet, X3-local store, X4-wholesale, X5-farmer, X6-marketplace, Age: 19–25 years, 26–40 years, 41–60 years, more than 60 years, place of living: RA-rural, C100-city with up to 100,000 residents, C100–300-city with 100,000–300,000 residents, C > 300-city with more than 300,000 residents, Education: Secondary education, Higher education, Professional education.

The classification tree distinguished consumers by gender (Figure 4a,b), who were divided into differentiated groups, making it possible to classify where they shop in relation to the other variables.

Figure 4a shows that three rules were created for such classifications. The final node (ID = 3) included consumers—women in the 19–25 and 41–60 years age groups—who shopped in a hypermarket and a local store.

The next node (node ID = 4) identified women aged 26–40 years living in small towns with up to 100,000 inhabitants and in large cities with more than 300,000 inhabitants who shopped online. 

The women assigned to the last node (node ID = 5) were those aged 26–40 years, living in rural areas and shopping mainly in hypermarkets and local stores.

In Figure 4b, four classification rules were created. The final node (ID = 4) consisted of consumers—men aged 26–40 years, who had a secondary or higher education and declared that they shopped online.

The next node (ID = 5) also identified a group of men, aged 26–40 years, who had a vocational education, and declared that purchases were made by cash and carry wholesalers.

In the case of node (ID = 8), it was this group that distinguished men aged 19–25 and 41–60 years and living in towns of 100–300,000 and over 300,000 that shopped at hypermarkets.

The last node (ID = 9) created a rule according to which men aged 19–25 years and 41–60 years, living in a rural area and in a small town (up to 100,000 inhabitants) declared shopping in hypermarkets and local stores.

Next, the focus was on a selected segment of food products, namely local products. Hence, in the next step, the results were analysed in terms of the availability of local products as perceived by food consumers.

**Figure 5:**
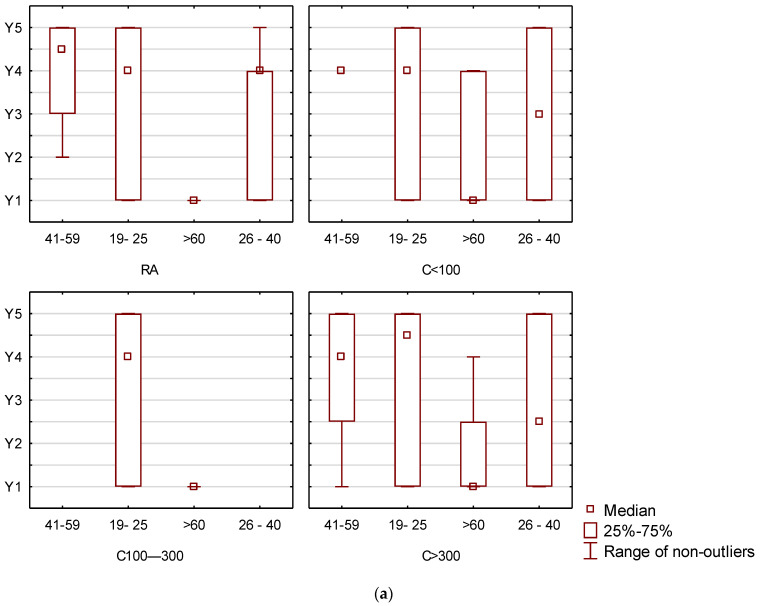

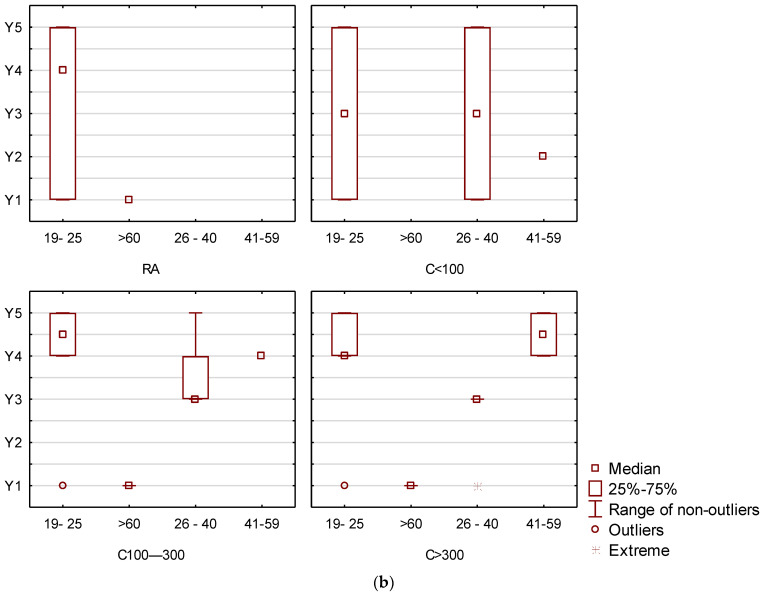
(**a**). Box plots for availability of local products among women’s responses by age and place of residence. (**b**). Box plots of local product availability among male responses by age and place of residence. Abbreviations: Y1-hypermarket, Y2-via internet, Y3-local store, Y4-wholesale, Y5-farmer, Y6-marketplace, Age: 19–25 years, 26–40 years, 41–60 years, more than 60 years, place of living: RA-rural, C100-city with up to 100,000 residents, C100–300-city with 100,000–300,000 residents, C > 300-city with more than 300,000 residents, Education: Secondary education, Higher education, Professional education.

Figure 5a shows the most frequently declared places of availability of local products among female consumers by the variables of age and place of residence. This segmentation was carried out based on the most statistically significant grouping variables contributing the most to the classification.

Women living in rural areas in the age groups 19–25 and 26–40 years indicated that the availability of local products varies, but that they are mainly available at cash and carry wholesalers. Women aged 41–59 years also indicated cash and carry wholesalers, but also farmers. In contrast, women over 60 years decided that products of a local nature can only be purchased at a hypermarket.

On the other hand, women living in small towns (up to 100,000 inhabitants), among them those aged 19–25 years, also decided that they buy local products in various places, but mainly at cash and carry wholesalers. Women aged 26–40 years declared that local products can be bought from a farmer, while those aged 41–59 years considered that they were exclusively cash and carry wholesalers. In contrast, women aged 60 years and over indicated mainly hypermarkets.

In the case of women living in medium-sized cities, i.e., 100–300,000 it can be observed that women aged 19–25 years declared that local groceries are mainly available at cash and carry wholesalers; while women over 60 years said they are available in hypermarkets.

The last group is made up of women living in large cities (over 300,000 residents). Therefore, women aged 19–25 years indicated that local products are mainly available at cash and carry wholesalers and from the farmers. Women aged 26–40 years indicated the Internet and local stores as places to purchase them, while women aged 41–59 years indicated cash and carry wholesalers. Women over 60 years indicated, as in previous groups, hypermarkets.

As can be observed by analysing the above-mentioned data, women of different ages (young and middle-aged) and living in demographically diverse areas see the greatest availability of local products in cash and carries and possibly farmers. Only young women living in large cities point to online shops and local stores. It is also noteworthy that older women, aged 60+ years, regardless of where they live, indicate that the greatest availability of local food products is in super and hypermarkets.

In turn, Figure 5b shows the most frequently declared places of availability of local products among male consumers by the variables of age and place of residence.

Men living in rural areas in the 19–25 years age group indicated that the availability of local products varies, but that they are mainly available at wholesales stores. In contrast, men over 60 years decided that products of a local nature can only be purchased in a hypermarket.

On the other hand, men living in small towns (up to 100,000 residents), including those aged 19–25 and 26–40 years, also considered that local products can be bought in various places, but mainly in local shops. In contrast, men aged 41–59 years decided that they were only available online.

In the case of men living in medium sized cities, i.e., 100–300,000, those aged 19–25 years admitted that local food products are mainly available at cash and carry wholesalers, as well as from the farmer. Men aged 26–40 years indicated the availability of such products at local stores. On the other hand, men over 60 years claimed that the greatest availability of the analysed products is at the hypermarket.

Men living in large cities (over 300,000 residents), is the last group discussed. In this group, men aged 19–25 years indicated that local products are available at cash and carry wholesalers and from the farmers, those aged 26–40 years indicated local stores as the place to purchase them, while men aged 41–59 years indicated cash and carry wholesalers and farmers. Men over 60 years indicated, as in previous groups as well, the greatest availability of local products in hypermarkets.

As can be seen from the above data, men are more diverse in their declarations regarding the places of greatest availability of local products. This is because they point to both cash and carry outlets, local stores, superstores and hypermarkets, as well as farmers and online stores.

## 5. Discussion

Similar studies on the preferred and declared places of purchase of food products, chosen by contemporary consumers, have been carried out by many other authors, in different countries or economic regions, reaching varying and often different conclusions.

Liu and Niyongira analysed consumer food shopping behaviour in China. The results of a survey of 1015 consumers in Nanjing and Beijing showed that consumers preferred to buy food from a supermarket [64]. According to Hawkes [65], supermarkets have an advantage over traditional retailers mainly in terms of the variety of food offered, the possibility of greater price reductions and a diverse shopping atmosphere.

Similar findings were also obtained in our own research, as it turned out that both women and men, living in areas of different population density, shop for food mainly in super and hypermarkets. The only exceptions are men—residents of small towns—who declared that they shop online and at large cash and carry wholesalers. This may be because, as other researchers have found, residents of rural areas and small towns often must travel longer distances when they want to use a supermarket to do their food shopping. This is due to inadequate infra-structure in such areas. As Debela and his team demonstrated [66], the distance to the nearest supermarket is significantly correlated with the share of supermarket purchases—greater distance is associated with a lower likelihood of using a supermarket and a lower share of food purchases at this type of retail stores.

It should be added, however, that in many countries, especially developing ones, supermarkets are growing faster, at the expense of more traditional markets and grocery stores, which may affect the pattern of results obtained. However, there is no doubt, that for many different economies of the world, such retail stores have become the most important supplier of food products [67,68].

In turn, Turčínková and Kalábová [69] have completed a study of Moravian consumers’ preferences while buying food. Their results suggest that consumers prefer retail stores with fresh food and a wider assortment. In addition, their choice is also influenced by convenient location—as the most preferred retail stores are those closest to the respondents’ place of residence or workplace. The authors also add that consumers are characterized by a certain level of loyalty, as most of them have their favourite store, where they usually do most of their grocery shopping.

This is partly consistent with the results of our research, as respondents also indicated neighbourhood shops as the second most frequent place to buy food products.

All the women participating in the study, living in areas with different population densities, as well as men living in small, medium, and large cities, also indicated online platforms and shops as another place for food shopping, but men with less frequency than women. From an in-depth analysis using the classification tree method, it can be concluded that these were particularly women aged 26–40 years living in small and large cities.

Therefore, regarding the distribution channel for food products such as the Internet, the results of studies obtained by the Dang et al. can be cited. They concluded that women are more likely to purchase such products online. This may be due to their roles in traditional families, where women bear most of the responsibilities related to household duties, especially meal preparation and grocery shopping. Indeed, using this purchase channel can help them be time efficient [70,71].

As important a variable as place of residence and gender is also the age of consumers. This was demonstrated in a study carried out by Massaglia with his team [72]; this is because they proved that among the discriminating factors affecting consumer preferences and behaviour, and where the consumer prefers to buy products, age is foremost.

The study conducted by Nguyen et al. [73] examined the purchasing habits of 549 individuals in Ho Chi Minh City, Vietnam. The research found that a preference for locally made goods was positively correlated with ethnocentric consumer attitudes. Additionally, it was discovered that older consumers were more inclined to have ethnocentric tendencies and favour local products, while younger consumers were not affected by ethnocentric attitudes. These results have important implications for both domestic and foreign businesses advertising their products in Vietnam. It can be observed that age will have a significant impact on consumer purchasing decisions.

A similar trend was noticed by the team of Aprile et al. [43]. Older consumers chose local products to sustain the national economy and believed that local food is tastier and is produced in their own region. However, young people are also increasingly willing to buy goods that have been produced in their home country. In addition, they pay attention to whether a given product was created in environmentally friendly conditions. For example, women aged 18–29 years are interested in buying environmentally friendly food products and consider “environmental impact” to be the most important attribute when buying local food.

In turn, the second part of the conducted research focused on the availability of local food products in the opinion of consumers, inhabitants of Eastern Poland.

Comparing the obtained results with those described by other authors, it is important to note again their diversity and often dissimilarity. This may be due primarily to cultural, social, or demographic differences.

Turčínková and Kalábová concluded that the origin of food plays an important role in consumers’ purchasing decisions. However, they added that there was no statistically significant relationship between the level of preference and the gender or size of the respondents’ locality of residence. On the other hand, a moderately strong relationship can be found between the selected values of local food and the age and education of respondents [69].

Hence, other authors have reached different conclusions. Hidalgo-Milpa et al. found that gender plays an important role in relation to the decision to purchase and place of purchase local food products, as women are the most likely to purchase local products in an effort to ensure the well-being and health of their family members [74].

In our own research, gender differences were observed only among residents of small towns, in almost all age groups. Very young women aged 19–25 years declared the greatest availability of local food products in cash and carry wholesalers, while men of the same age declared the greatest availability in local neighbourhood shops. Younger female consumers (26–40 years) indicated buying directly from farmers, while men in the same age group declared shopping in neighbourhood shops. Differences could also be seen for middle-aged people, with women preferring the cash and carry wholesale shops option and men preferring online shops and platforms.

As can be seen from the above, not only gender, but also age and place of residence are important for the analysis and assessment of consumer attitudes. Furthermore, Brown had already proved in 2003 that attitudes toward local food depend on the origin of the respondents [14]. Furthermore, Bimbo and his team showed that age, education and occupational status positively correlate with high frequency of local food purchases [75]. Therefore, it may follow that the variables used for the analysis in this study have been selected correctly.

The increasing demand for local food products from year to year, together with a higher level of consumer awareness (in the areas of ecology, sustainable consumption or food safety and quality) are leading to interest and, consequently, the creation of shorter and shorter supply chains. Consequently, farmer’s markets and direct sales from the farm or consumer cooperatives (food cooperatives) are becoming increasingly popular places to buy such products [17,76]. This is because they generate a wide variety of benefits, including improved producer-consumer communication systems, bringing consumers closer to food sources (shortening producer-consumer distances), and increasing the reintegration of agriculture into more environmentally sustainable types of production, among others [77,78]. An additional advantage is that the shorter the supply chain, the easier it is to maintain the authenticity and originality of the food product. Furthermore, among consumers participating in the survey, some interest in this type of place of purchase (purchasing local food products at local stores or directly from farmers) was noted.

In turn, referring to studies conducted in Poland, Bryla’s work should be mentioned. After analysing the results, he found that Polish consumers preferred to buy regional products in producer-owned stores rather than in large distribution chains. They were also characterized by attitudes of national ethnocentrism towards the regional food market [79]. 

The research conducted among the inhabitants of Eastern Poland only partly coincided with the results obtained by Bryla [79], as in the presented study respondents referred to both local shops and large sales outlets. At the same time, young consumers indicated with greater frequency local shops or purchases directly from farmers.

As mentioned earlier, in Poland, the sale of food products is mainly carried out using traditional distribution channels, such as purchases in super- and hypermarkets, in local or neighbourhood stores, at local markets or directly from farmers. Although new-modern distribution channels for such products, such as online shops or platforms, are not yet very popular, their rapid and dynamic development can be noted, due to the growing interest of contemporary consumers in such forms of shopping.

Barska and Wojciechowska-Solis conducted detailed research in this area in Poland. The authors, analysing online purchases of local products, found that the group of e-consumers was dominated by women, younger people, and residents of large cities. In addition, they observed significant differences between consumers purchasing such food products in stationary local grocery stores (such as stalls, markets, stores at farms selling their own products) and the total sample due to certain sociodemographic variables describing them [80]. The results obtained by Barska and Wojciechowska-Solis were partly confirmed in our own research. The greatest availability of local groceries of online shops, and consequently purchases through this distribution channel, were indicated mainly by young women living in large cities, but also by middle-aged men living in small towns. 

It should be added that the results obtained by Fernández-Ferrín et al. [81] have confirmed that there is a fairly significant correlation between the motives that Spanish researchers have identified as ethnocentric in consumers and the propensity to buy local products. This is because tradition, combined with the geographical origin of the product, is an important component of local identity. As a result, this may induce consumers to buy local products, especially because they will simultaneously be able to support local development, as well as improve the financial situation of the local community [82].

The above findings were also confirmed by Memry and his team, arguing that the justification for consumers to purchase local products is not the intrinsic quality of the products, but the desire for local support. The authors added that this support has a stronger effect when local identity is higher [15].

## 6. Conclusions

Consumer behaviour is a very difficult and complex topic to consider, not least because it consists of all the thoughts, feelings, and actions involved in choosing a product and buying it. Factors influencing these variables are mainly gender, age or place of residence. Purchasing decisions will vary considerably according to these parameters.

Hence, the aim of the research conducted was analysis of consumer behaviour in the context of the place of purchasing food products with particular emphasis on local products. In addition, an attempt was made to analyse the perceived availability of local products based on three grouping variables: gender, age and place of residence. The research was conducted among the inhabitants of Eastern Poland, particularly in the Lubelskie Voivodeship. 

Referring to the research questions it should be noted that:

1. Both women and men, living in areas with different population density, buy food products mainly in super- and hypermarkets, which is consistent with the results obtained by other authors conducting research in different countries [64,65].
2. Only male residents of small towns declared that they purchase online and at large cash and carry wholesalers. On the one hand, this may be due to longer distances to large supermarkets, and on the other hand, to insufficient infrastructure in such areas. Similar conclusions were also reached by Debela and his team [66].
3. As the second place for the most frequent purchases of food products, respondents also indicated local shops. This may be related to the convenient location of such places, or habit or loyalty. This may also be because local retailers offer fresher food in the opinion of consumers. The gained results confirm the results obtained by Turčínková and Kalábová [69].
4. Buying food products via online platforms and shops is mainly the domain of young and middle-aged people, especially those living in large cities—all women, living in areas with different population densities, and men—residents of small, medium, and large cities, indicated online platforms and shops as the place where they purchase their food products.
5. Gender does not always differentiate consumers’ perception of the availability of local products. This was only evident among residents of small towns, in almost all age groups. The obtained results were only partially consistent with the results reported by other authors. This may be primarily due to cultural, social, or demographic differences.
6. Respondents’ perceived availability of local food products also varied slightly in relation to age. Differences could only be observed among younger consumers, who were more likely to indicate neighbourhood shops or buying directly from farmers. Other respondents declared that local food products could be purchased in both local shops and large retailers. The results obtained did not confirm the conclusions drawn by other authors such as Bryla [79].
7. In Poland, the sale of local food products is mainly carried out using traditional distribution channels, such as purchases in super- and hypermarkets, in local shops, at local markets or directly from farmers. However, contemporary consumers are also becoming increasingly interested in modern distribution channels for these types of products, such as online shops or platforms. The study found that the greatest availability of local food products of online shops, and thus purchases through this distribution channel, were indicated mainly by young women living in large cities, but also by middle-aged men living in small towns, which largely confirms the results obtained by Barska and Wojciechowska-Solis [80].

Despite the local character of the conducted research, the contribution seems to be important. In the region of Eastern Poland, there is a limited amount of research in the context of places of purchase of food products considering the consumers’ gender, age, and place of residence. In addition, the obtained research results can be an important starting point for further in-depth analysis and research, especially in the field of consumer attitudes and preferences regarding the purchase of local products, and the phenomenon of ethnocentrism.

Importantly, the influence of various variables on consumer purchasing decisions is certainly worthy of empirical research and systematic analysis in the future due to the changing market environment, product availability and variety of offerings and, crucially, consumer preferences. Furthermore, such a strategy may be essential for the development of appropriate marketing activities by the various companies involved in the sale of food products, both retail and wholesale. It should be emphasized that this study does not cover the entire complex process of purchasing food products, hence it is advisable to conduct further research related to the issue of consumer behaviour.

## Figures and Tables

**Table 1:** Consumer research in Eastern Poland—basic characteristics.

Description	Characteristic
Research objectives	− to collect research material to enable the segmentation of consumers according to specific determinants of their food purchasing behaviour by product origin, − to identify consumer attitudes and to show how these attitudes translate into purchasing habits in relation to different grouping variables.
Research object	individual consumers of food products
Type of research	qualitative research
Research method and technique	survey distributed via Internet
Research tools	proprietary survey questionnaire
Selection of units for research	non-random, targeted
Sample selection criteria	individual consumers, a group differentiated by sex, age, place of residence
Sample size	404 persons
Spatial scope	Eastern Poland
Time range	January–June 2022

## Data Availability

Not applicable.
